# Supplementary material for: Emergent layer stacking arrangements in c-axis confined MoTe2
Source: Nat Commun. 2023 Aug 9;14:4803. doi: 10.1038/s41467-023-40528-y (PMC10412583; doi:10.1038/s41467-023-40528-y)
Supplement: Supplementary file 1 — Supplementary Information [file 41467_2023_40528_MOESM1_ESM.docx]

**Emergent Layer Stacking Arrangements** **in *c*-axis Confined MoTe_2_**

SUPPLEMENTARY INFORMATION

James L Hart^1^, Lopa Bhatt^2^, Yanbing Zhu^3^, Myung-Geun Han^4^, Elisabeth Bianco^5^, Shunran Li^6,7^, David J Hynek^7,8^, John A Schneeloch^9^, Yu Tao^9^, Despina Louca^9^, Peijun Guo^6,7^, Yimei Zhu^4^, Felipe Jornada^10^, Evan J Reed^10^, Lena F Kourkoutis^2,5^, Judy J Cha^1,11*^

*1. Department of Materials Science and Engineering, Cornell University, United States*

*2. School of Applied and Engineering Physics, Cornell University, United States*

*3. Department of Applied Physics, Stanford University, United States*

*4. Condensed Matter Physics and Materials Science Department, Brookhaven National Laboratory, United States*

*5. Kavli Institute at Cornell for Nanoscale Science, Cornell University, United States*

*6. Department of Chemical and Environmental Engineering, Yale University, United States*

*7. Energy Sciences Institute, Yale University, United States*

*8. Department of Mechanical Engineering and Materials Science, Yale University, United States*

*9. Department of Physics, University of Virginia, United States*

*10. Department of Materials Science, Stanford University, United States*

*11. Cornell Center for Materials Research, Cornell University, United States*

*Corresponding author: jc476@cornell.edu

**Supplementary Note 1: Accuracy of Te-Te bond mapping**

To extract the Te-Te shifts across the vdW gap, we fit each Te column with a 2D Gaussian. To evaluate the accuracy of this methodology, we consider STEM images down the [100] axis, as shown in Supplementary Figure 2. Along this axis, the expected inter-layer shift is 0 pm for both 1T´ and T_d_ stacking. Hence, our analysis should yield shifts of 0 pm, and any measured shift may be interpreted as the inherent error in our approach. As shown in Supplementary Figure 2, the average shifts across a given layer are ≤ 10 pm, and within a given layer, the standard deviation of the measured shifts is also ≤ 10 pm. This is true for both bulk and thin flakes of MoTe_2_. We thus claim a measurement uncertainty of ≤ 10 pm.

**Supplementary Note 2: Simulated HAADF-STEM images**

The simulation structures in Supplementary Figure 4 were created based on the twin fault and mixed 1T´-T_d_ state disorder observed in the cross-sectional experimental imaging (Fig. 3). For optimal plan-view imaging the projection direction should coincide with a high-symmetry zone axis. For 1T´ and T_d_ MoTe_2_, for example, the crystalline *c*-axes are tilted by ~3.9° with respect to each other as indicated in Supplementary Figure 4a,b. For each simulated disordered structure, we similarly determined the appropriate tilt angle and accounted for it by tilting the structure for simulation. As seen from Supplementary Figure 4, a variety of structures are observed in the simulated plan-view HAADF-STEM images which can help explain the variety of structures seen in the experimental plan-view images.

**Supplementary Note 3: Stacking models**

Crystal structures for the 1T´ and T_d_ phases were taken from ref. 11 of the main text. For the random stacking model (see Figs. 4 and 5 of the main text), we started with the single-layer structure of the 1T´ phase, and then constructed the crystal layer-by-layer. For each new layer added to the structure, the shift direction was randomly selected, with the shift magnitude fixed at 0.45 Å. For reference, in the bulk 1T´ phase, the shift magnitude is 0.46 Å, and for the T_d_ phase, the shift magnitude is 0.36 Å. These values are based on bulk diffraction data from ref. 11. Note that in our STEM images, there is evidence for local variations in the magnitude of **λ**. Incorporating variation in the magnitude of **λ** in our simulations of 1T´ and T_d_ leads to some diffuse scattering and broadening of Bragg spots along *L*. This effect, however, is minimal compared to the diffuse scattering generated by randomly varying the direction of **λ** in the random stacking model. For the T_d_-Rnd structure, each new layer added to the structure had an 85% likelihood of following T_d_ stacking, based on the prior layer. For instance, if the prior layer shift was ↓, the next shift had an 85% likelihood of being ↑. This method produced a disordered structure with local T_d_ stacking, having an average T_d_ domain thickness of ~ 4 nm.

**Supplementary Note 4: Quantitative electron diffraction**

Multi-slice simulations were performed with the Computem package from Earl Kirkland. Since the non-orthogonal unit cell of the 1T´ phase prevents the use of periodic boundary conditions, all simulations were performed on cylindrical nanoparticles, with a diameter of 45 nm and a thickness of 80 nm, embedded within a simulation cell of 50 × 50 × 82 nm. For the simulations, the real space pixel size was 0.005 nm, the reciprocal space pixel size was 0.02 nm^-1^, and the slice thickness was 0.25 nm. For each slice of the calculation, the wavefunction was saved, allowing diffraction patterns to be determined for all thicknesses. Calculations were performed at 0 K (no frozen phonon configurations were used). For each stacking sequence, simulations were performed for misorientations about the *a-* and *b*-axes, α and β, of ±1.5°, using a step size of 0.1°. To account for flake bending, we locally averaged together the tilt-dependent simulations using a Gaussian kernel:

Equation 1: $I_{sim}=\sum_{\alpha} \sum_{\beta} {I(z)}_{\alpha,\beta}\times\exp\left( \frac{\left( \alpha-\alpha_{0} \right)^{2}\left( \beta-\beta_{0} \right)^{2}}{\gamma} \right)$

where *I*_sim_ is a vector containing the 76 Bragg spot intensities, *I*(*z*)_α,β_ represents the spot intensities from a single multi-slice simulation of a certain thickness and tilt, α_0_ and β_0_ are the center of the Gaussian weighting function and physically represent the average orientation of the flake, and γ controls the width of the Gaussian and physically corresponds to the degree of flake bending. The fits were performed in python using the scipy curve_fit module, and evaluated using the χ*_v_*^2^ metric:

Equation 2: $\chi_{v}^{2}=\frac{\sum\left( \frac{I_{exp}-I_{sim}}{\sigma_{exp}} \right)^{2}}{n-m}$

where σ_exp_ is the experimental error associated with each Bragg beam, and *I*_exp_ and *I*_sim_ were both normalized to integral unity. The denominator represents the number of degrees of freedom *v*, given by the number of datapoints *n* minus the number of fitting parameters *m*. Note that for calculation of *I*_sim_ and χ*_v_*^2^, each (*H*, *K*, 0) spot is treated independently, while for presentation within the text, symmetry related (*H*, *K*, 0) spots are averaged together for ease of viewing. Note that σ_exp_ has two components, one related to background subtraction for each individual diffraction spot, and a second related to merging of different acquisitions (as discussed below).

To acquire the diffraction data, we used a CCD camera, where the dynamic range was not sufficient to capture all diffraction spot intensities simultaneously. Thus, we performed multiple acquisitions for each diffraction pattern using various exposure times, usually 0.1, 1, and 10 s, with the shorter acquisitions needed to avoid saturation of the most intense spots, and the longer acquisitions needed to accurately measure the weaker spots. To merge the three datasets together, diffraction spots of moderate intensity (those which were not saturated in the longest exposure but still observable for the shortest exposure) were selected. Using these moderate intensity spots, we fit a scaling factor to the 0.1 and 1 second exposure datasets to minimize the relative differences between all three datasets. The relative error in merging the datasets was around 10%.

Lastly, we note that for the disordered stacking model, different stacking sequences were used for each of the different tilts. Thus, when averaging together different tilts to account for flake bending (Equation 1), we are also averaging over dozens of distinct stacking sequences, each randomly generated. Hence, while each randomly generated stacking sequence will yield slightly different diffraction spot intensities, the computed *I*_sim_ will reflect the average diffraction signal from disordered stacking.

**Supplementary Note 5: Exfoliation procedure**

With our exfoliation method, many adjacent 2D crystals of MoTe_2_ are placed on scotch tape, and then repeatedly ‘copied’, by transferring to a new section of the scotch tape. Each copy reduces the average MoTe_2_ flake thickness. For the final step, the scotch tape is pressed down onto a SiO_2_ / Si wafer. In this step, the force exerted on each flake is related to the applied downward force and is not dependent upon the flake thickness. Next, the tape is slowly peeled back. We assume that during this step, flakes which are vdW bonded to the SiO_2_ substrate and the scotch tape are ripped in half, with the bottom half left on the SiO_2_ substrate, and the other half remaining on the scotch tape. The thickness of the flake left on the SiO_2_ substrate – which is what we study experimentally – is randomly determined when the flake is split in half during this final step of exfoliation. Thus, there is no step during our experimental procedure where the flake thickness influences the applied stress or strain.

**Supplementary Note 6: Flake thickness determination**

For electronic transport and Raman spectroscopy (Fig. 1), the flake thicknesses were measured with atomic force microscopy. For the plan-view electron diffraction measurements (Fig. 5), flake thicknesses were measured with EELS. We note that in both cases, the measured flake thickness will include contributions from surface contamination and surface oxidation, if present. For cross-section STEM measurements (Figs. 3 and 4), the flake thickness was determined directly from the real-space STEM images, accounting only for the crystalline phase.

**Supplementary Figures:**


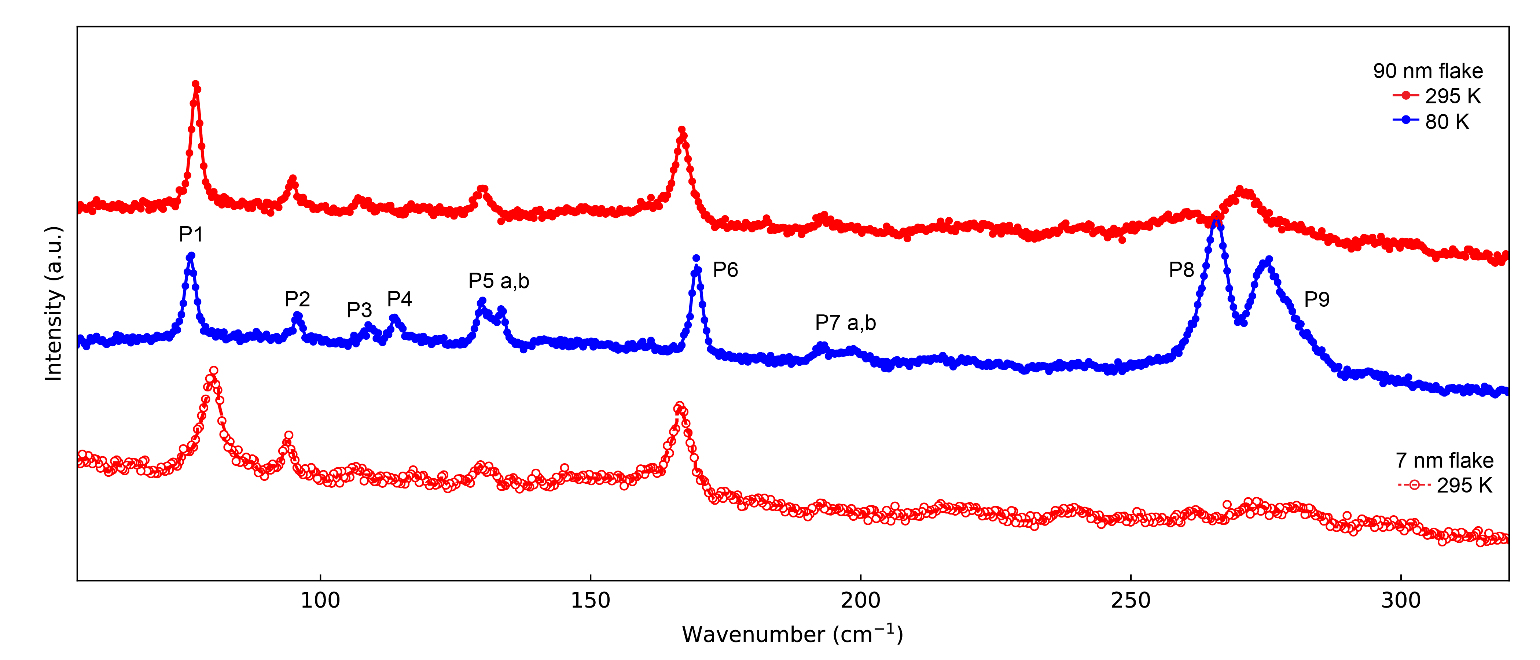


**Supplementary Figure 1 | Extended Raman spectroscopy data.** For the 80 K data, the Raman modes are labeled according to ref. 19.


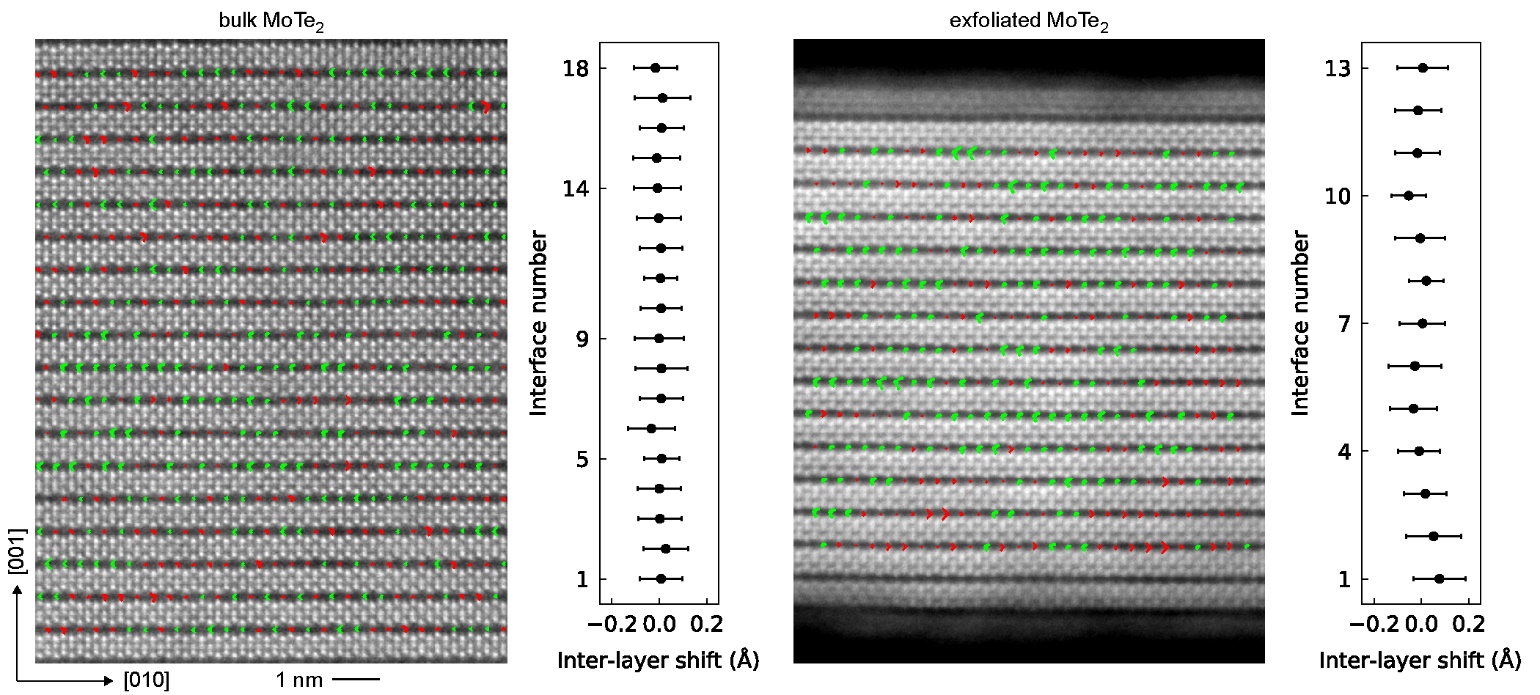


**Supplementary Figure 2 | [100] atomic-resolution imaging.** HAADF-STEM of bulk and thin flake MoTe_2_ imaged down the [100] zone axis. The in-plane displacement component for each individual Te-Te pair is represented with an arrow, whose magnitude is 15× the actual measured shift. Note that in this orientation, the 1T´ and T_d_ (and disordered) stacking models are equivalent, and the bridging Te-Te pairs should have no in-plane component. Consistent with this expectation, we find that the measured inter-layer shifts are quite small. To the right of each image, we plot the average measured shift for each layer. The average measured shift for each layer is < 10 pm, and the standard deviation within each layer (represented with the error bars) is also ≤ 10 pm. We interpret these measured shifts as the uncertainty within our analysis.


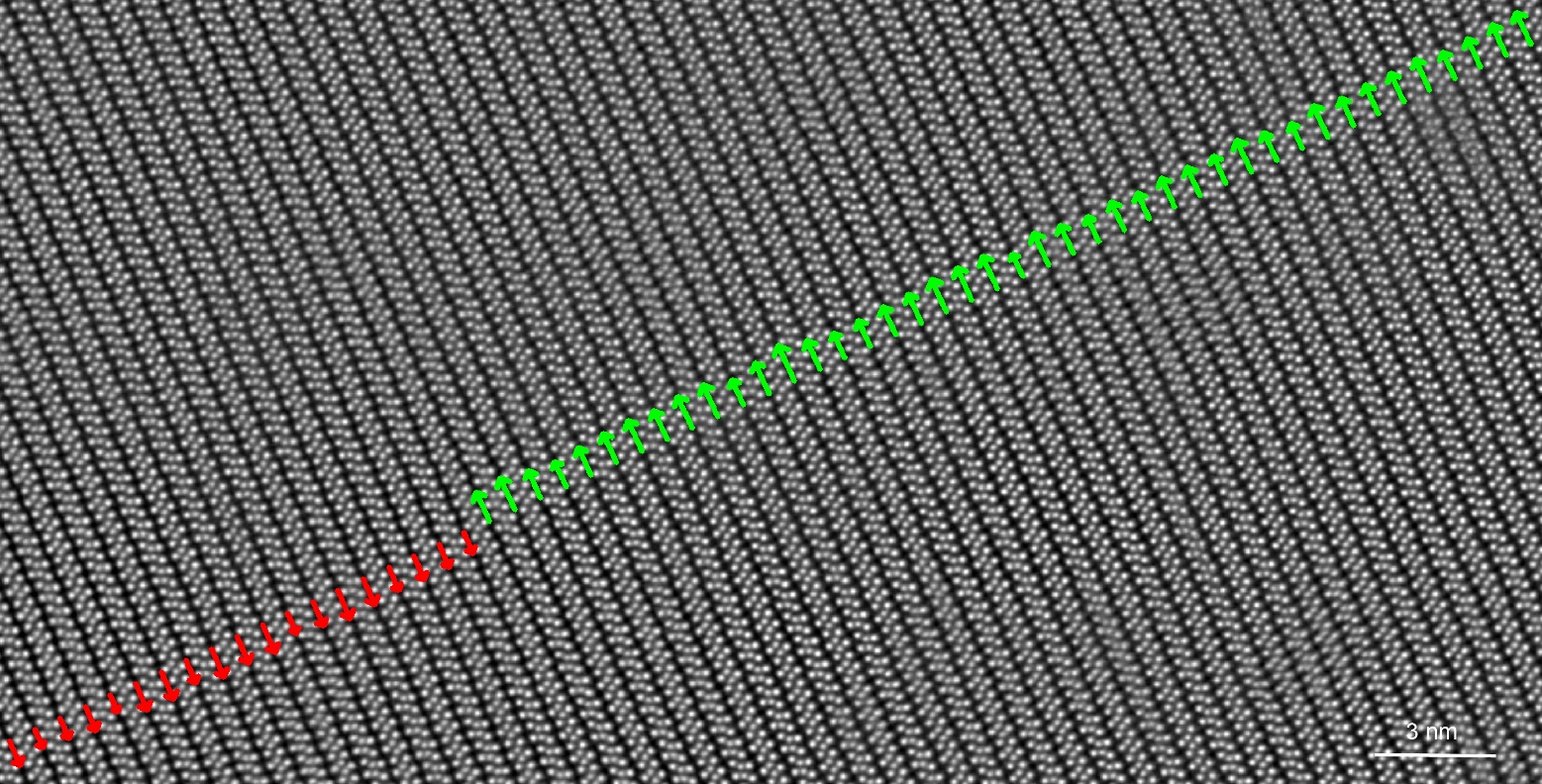


**Supplementary Figure 3 | Stacking defects in bulk MoTe_2_.** Example domain boundary in bulk 1T´ MoTe_2_ imaged by HAADF-STEM.

**
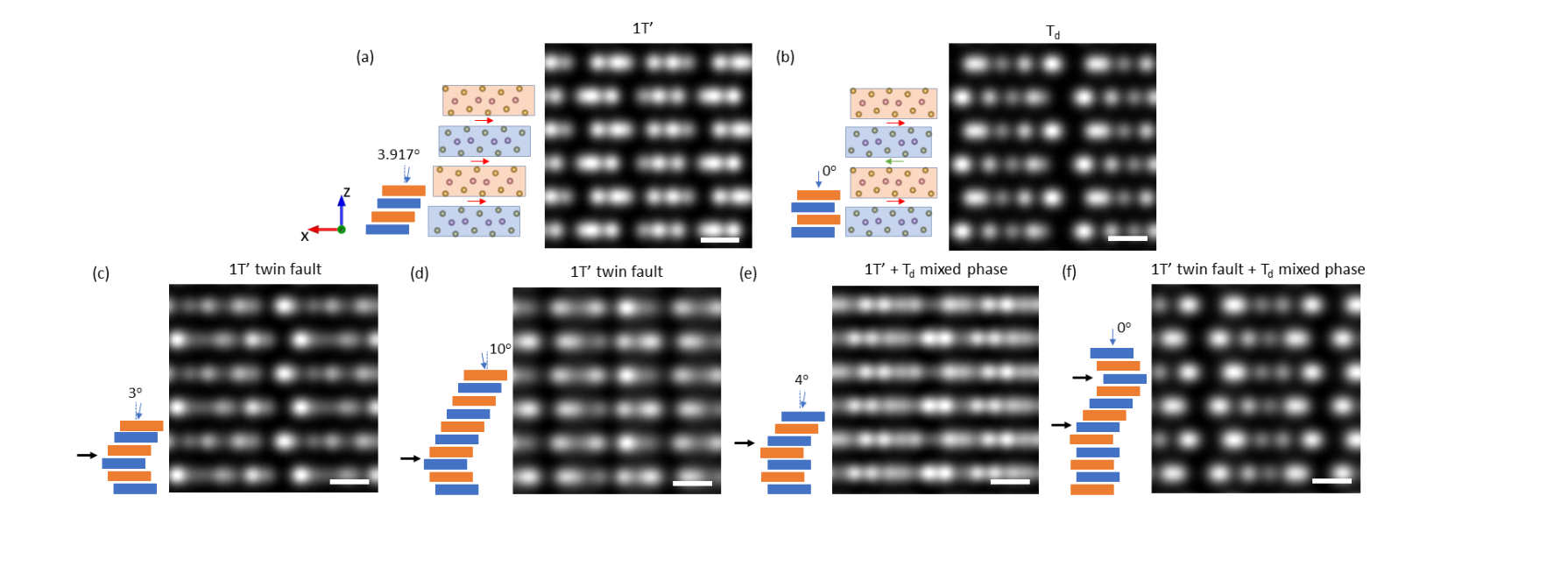
**

**Supplementary Figure 4 | Plan-view STEM simulations.** Simulated plan-view HAADF-STEM images of (**a**) 1T´ and (**b**) T_d_ MoTe_2_ (imaged down the *c-axis*). Included on the left are schematics of the layer stacking structure viewed in cross-section along the *b*-axis. (**c**-**f**) Simulated plan-view HAADF-STEM images of thin MoTe_2_ crystals with disordered layer stacking in the form of (**c, d**) 1T´ twin fault structures, (**e**) 1T´+T_d_ mixed structures and (**f**) 1T´+T_d_ mixed structures with a twin fault. The blue arrow in each schematic indicates the beam propagation direction for the simulations. The scale bar length is 2 Å for all images.


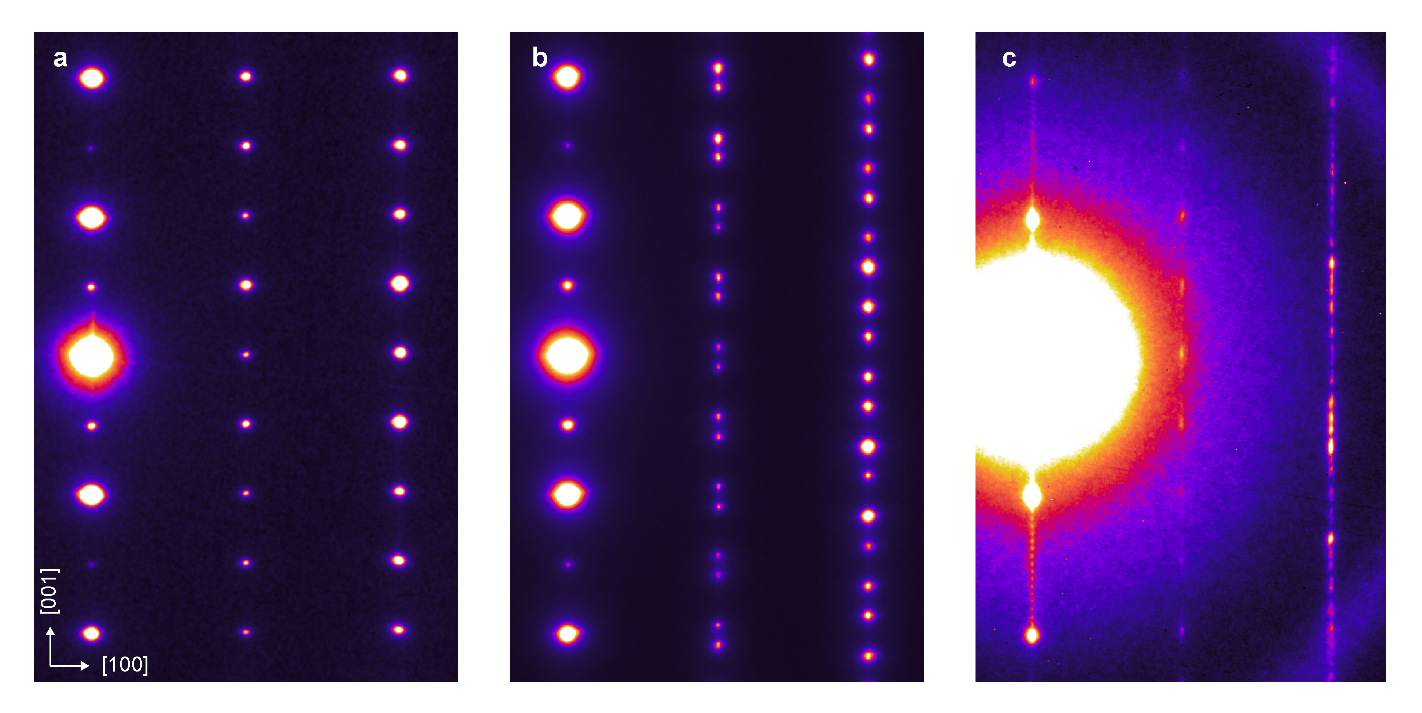


**Supplementary Figure 5 | Extended electron diffraction data.** Expanded *ac*-plane diffraction data for bulk T_d_ WTe_2_ (**a**), bulk 1T´ MoTe_2_ (**b**), and disordered 11 nm thick MoTe_2_ (**c**). In **c**, the amorphous ring from the underlying SiO_2_ substrate is observed.


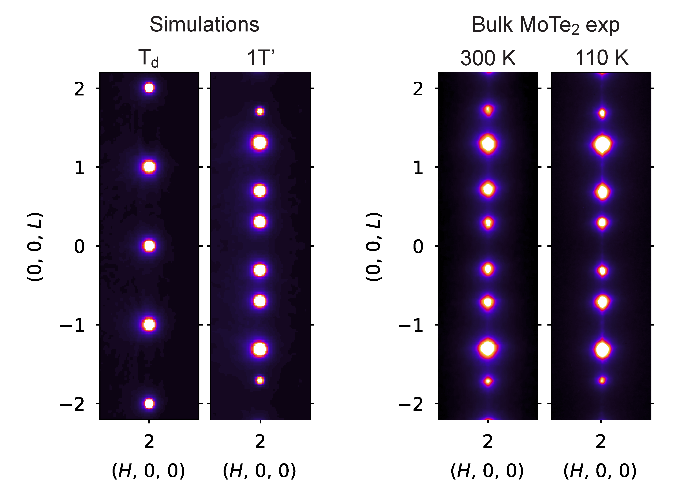


**Supplementary Figure 6 | Cryogenic cross-section electron diffraction.** *In situ* liquid nitrogen cooling of a bulk (> 5 μm thick) MoTe_2_ FIB lift-out. With cooling down to ~110 K, the 1T´ to T_d_ transition with *T*_c_ ~ 250 K is expected in bulk. Instead, the bulk lift-out shows minimal changes with cooling. This result suggests that FIB preparation locks the layers in place, preventing the layer sliding transition. We speculate that the outer amorphous layer created during FIB thinning locks the layers in place, suppressing the layer sliding transition.


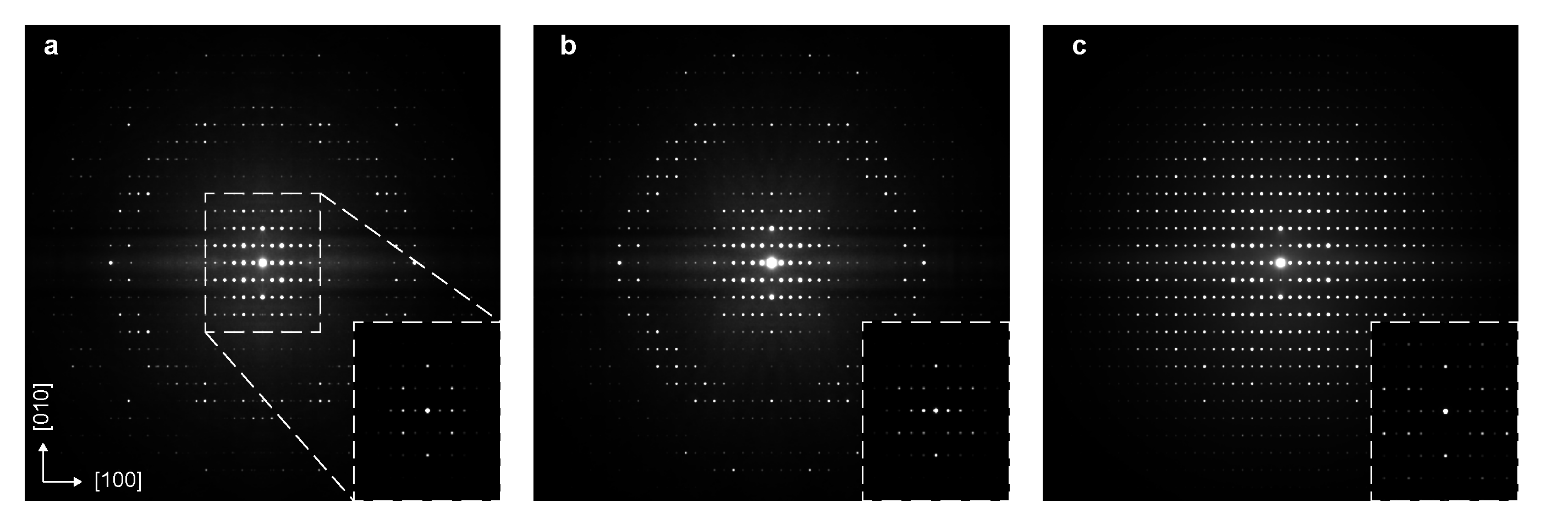


**Supplementary Figure 7 | Simulated plan-view electron diffraction of MoTe_2_.** Simulated electron diffraction data for MoTe_2_ with 1T´ stacking (**a**), T_d_ stacking (**b**), and disordered stacking (**c**). The simulation thicknesses were 35 nm. While the ordered structures show a first order Laue zone, the disordered structure does not. The lower right insets show the central region of each diffraction pattern with adjusted contrast limits, highlighting that the spot intensities vary as a function of stacking.


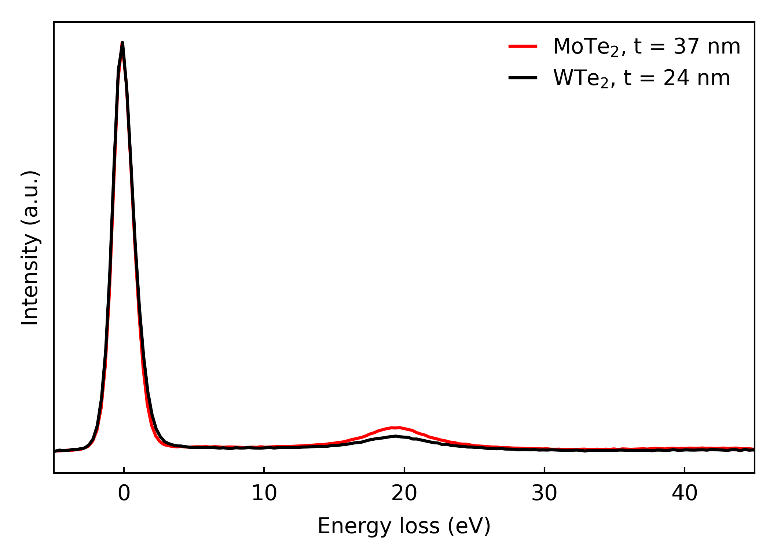


**Supplementary Figure 8 | EELS thickness measurements.** EELS zero loss peak data. For MoTe_2_, the measured thickness was 0.43 inelastic mean free paths, with a calculated mean free path thickness of 87 nm. For WTe_2_, the measured thickness was 0.29 inelastic mean free paths, with a calculated mean free path thickness of 82 nm. The figure legend provides the thickness (t) in nm. The inelastic mean free paths were calculated using David Mitchell’s Mean Free Path Estimator, a Gatan plugin.


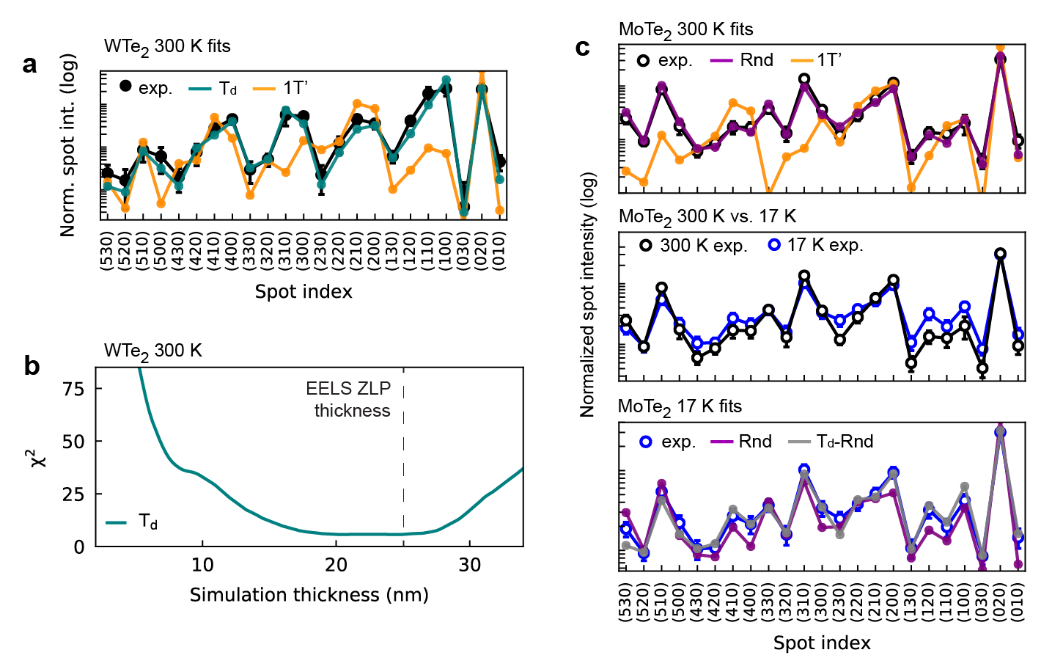


**Supplementary Figure 9 | Quantitative electron diffraction analysis. a.** Comparison of experimental and simulated diffraction data for WTe_2_. The T_d_ model provides a much better fit to the data. **b.** χ*_v_*^2^ for the WTe_2_ T_d_ fit plotted as a function of simulation thickness. The vertical dashed line shows the experimentally determined flake thickness (uncertainty is ± 5 nm). **c.** Comparison of experimental and simulated diffraction data for MoTe_2_ at both 300 and 17 K. For **a** and **c**, the plotted (*H*, *K*, 0) data points average together all symmetry related spots, *e.g.,* (*H*, *K*, 0), (-*H*, *K*, 0), *etc.*, but for the actual fitting procedure, these spots are treated independently (Supplementary Note 4). The χ*_v_*^2^ values for all the fits are displayed in the main text, Table 1.


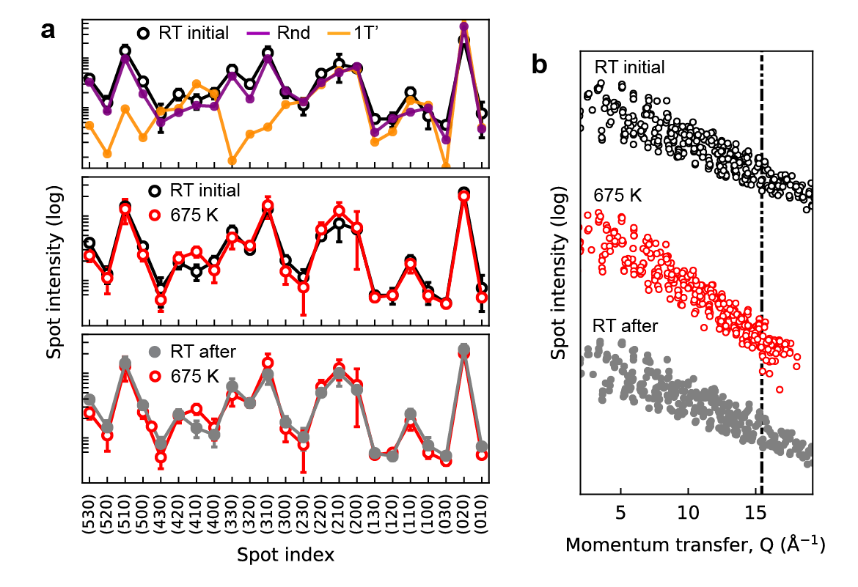


**Supplementary Figure 10 | Quantitative electron diffraction analysis with heating.** *In situ* annealing of a MoTe_2_ flake up to 675 K. The quantitative fits are shown in **a**, and the spot intensity as a function of *Q* is shown in **b**. Initially, the data at room temperature is well fit by the random stacking model, and there is no Laue zone, indicative of mixed / disordered stacking. With heating up to 675 K, and then cooling back down to room temperature, there are minimal changes in the measured diffraction spot intensities, and no appearance of a Laue zone. Hence, post-exfoliation annealing has no measurable effect on the layer stacking.


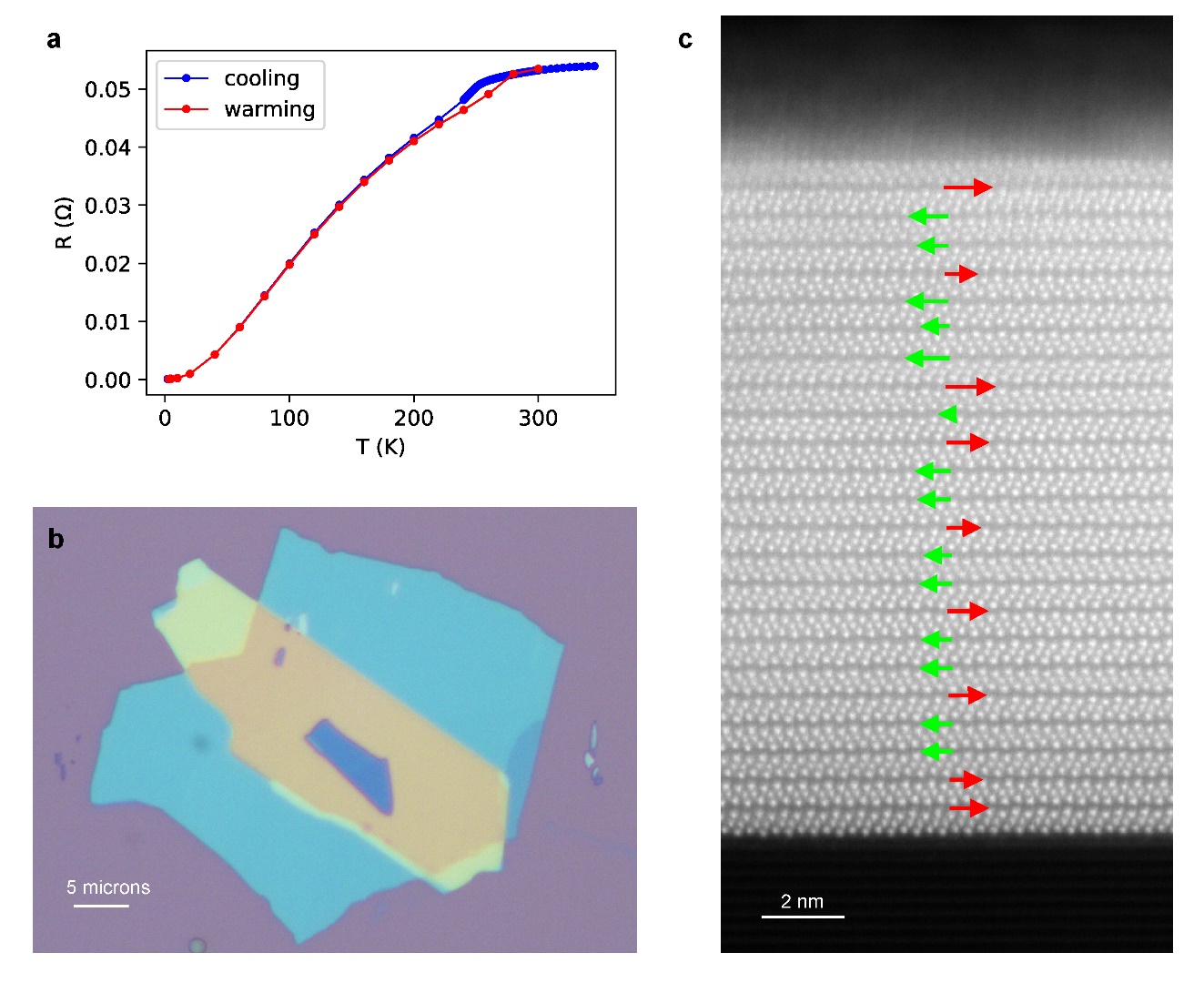


**Supplementary Figure 11 | Analysis of high RRR MoTe_2_ with h-BN encapsulation**. **a.** Example resistance versus temperature data for a crystal grown with the conditions described in refs. 30 and 33 of the main text. For this crystal, the RRR = 478. A separate crystal showed RRR = 797. **b.** Optical image of a MoTe_2_ flake with top and bottom hexagonal boron nitride encapsulation layers. Exfoliation and heterostructure assembly were performed in an Ar glovebox with O_2_ and H_2_O levels < 0.5 ppm, using MoTe_2_ crystals as described in refs. 30 and 33 of the main text. **c.** HAADF-STEM image of the same heterostructure, demonstrating the presence of layer stacking disorder.


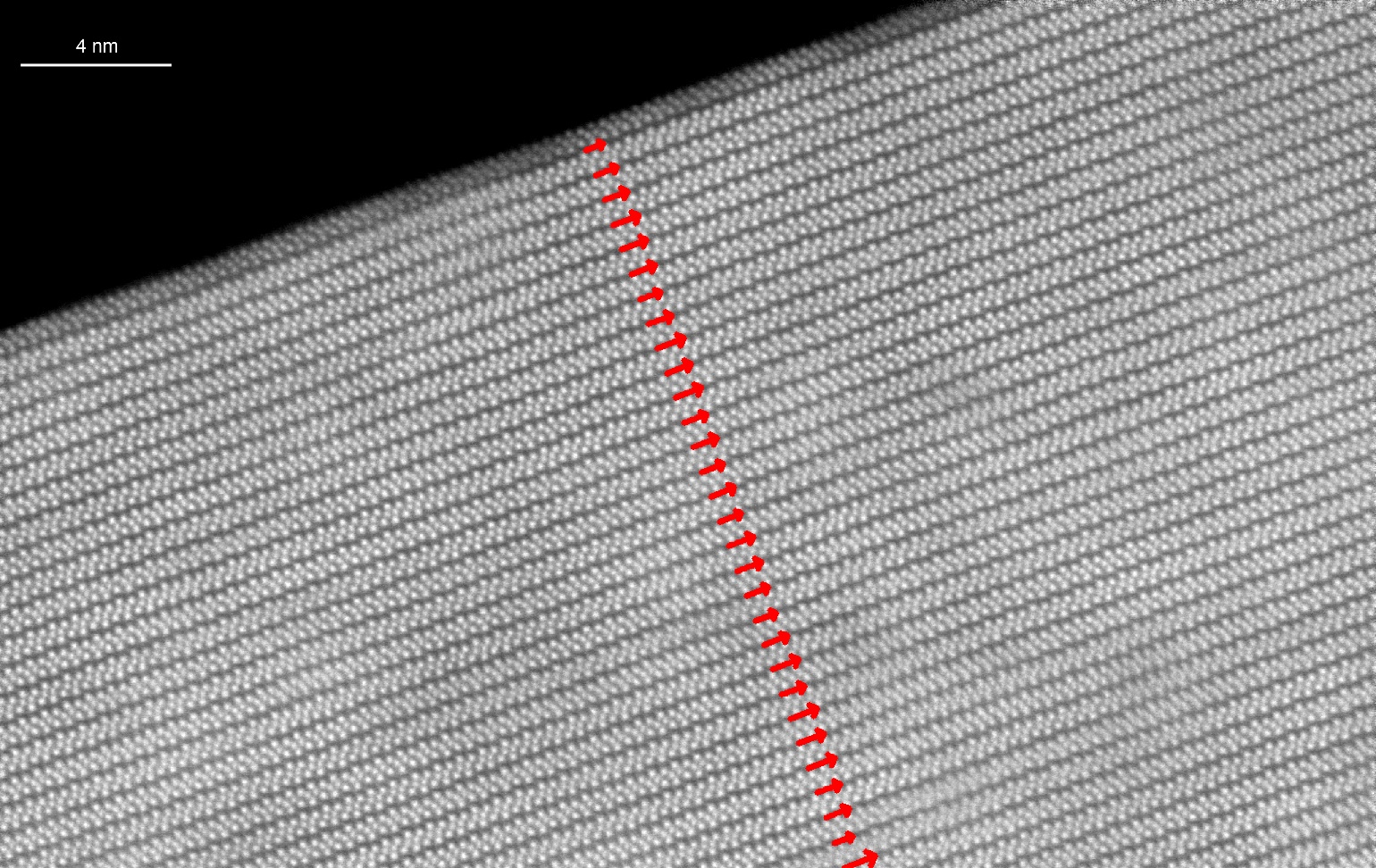


**Supplementary Figure 12 | Stacking analysis at the MoTe_2_ surface.** Stacking in MoTe_2_ at the top surface of a bulk crystal imaged by HAADF-STEM. The top surface of the crystal was freshly exfoliated prior to FIB sample preparation, and exposed to ambient atmosphere for several minutes.


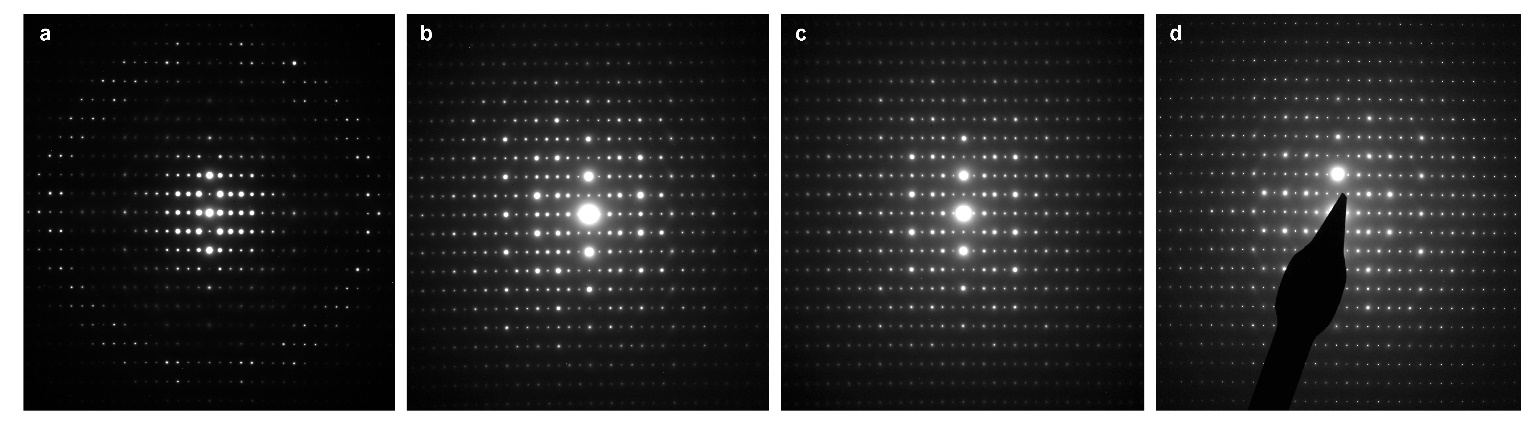


**Supplementary Figure 13 | Comparison of MoTe_2_ crystal sources.** *ab*-plane electron diffraction comparison of different crystal sources. **a.** Diffraction of WTe_2_ from 2D Semiconductors, showing a strong 1^st^ order Laue zone, indicative of ordered layer stacking. Diffraction of MoTe_2_ exfoliated from crystals obtained from 2D Semiconductors (**b**), HQ Graphene (**c**), and lab grown crystals as reported in refs. 30 and 33 of the main text (**d**). Note that none of the MoTe_2_ diffraction patterns show a Laue zone, indicating disordered layer stacking.

**Supplementary Table 1.** DFT computed energy difference between the T_d_ stacking and 1T´ stacking. The reported energies correspond to fully relaxed structures at 0 K. Bolded values correspond to structures where the 1T´ stacking is lower in energy than T_d_ (where ∆*E* is positive).

|  | Thickness  (# of layers) | *∆E* = *E*_Td_ – *E*_1T’_ (meV / formula unit) | | |
| --- | --- | --- | --- | --- |
|  |  | Grimmes | Rev | Rev+U |
| MoTe_2_ | Bulk | -0.42 | -0.11 | -0.27 |
|  | 5 | -0.12 | -0.14 |  |
|  | 4 | **0.29** | -0.20 | **3.23** |
|  | 3 | -0.21 | -0.14 | -256 |
|  | 2 | -0.22 | **0.01** | **0.66** |
| WTe_2_ | Bulk | -2.55 | -1.51 | -0.54 |
|  | 4 | **5.04** | **3.08** | **0.39** |
|  | 3 | -0.62 | -0.43 | **0.14** |
|  | 2 | -0.82 | -0.93 | -0.23 |
